# Reasons for defaulting from childhood immunization program: a qualitative study from Hadiya zone, Southern Ethiopia

**DOI:** 10.1186/s12889-016-3904-1

**Published:** 2016-12-09

**Authors:** Asamnew Zewdie, Mekitew Letebo, Tinsae Mekonnen

**Affiliations:** 1 Private Consultant, Addis Ababa, Ethiopia; 2 Clinton Health Access Initiative, Addis Ababa, Ethiopia

**Keywords:** Childhood immunization, Defaulter, Ethiopia, Health systems, Tracking

## Abstract

**Background:**

Reduction of mortality and morbidity from vaccine-preventable diseases in developing countries involves successfully implementing strategies that ensure high coverage and minimize drop-outs and missed opportunities. Achieving maximum coverage, however, has been a challenge due to many reasons, including high rates of defaulters from the program. The objective of this study was to explore the reasons behind defaulting from the immunization program.

**Methods:**

A qualitative study was conducted in two districts of Hadiya zone, Southern Ethiopia between November 2014 and April 2015. A total of twenty-six in-depth interviews were held with mothers of defaulted children aged 6-11months old and key informants from the communities, health centers, and health offices. Observations and review of relevant documents were also conducted. Thematic analysis was used to analyze the data.

**Results:**

In this study, the main reason for defaulting from the immunization was inadequate counseling of mothers that led to a lack of information about vaccination schedules and service arrangements, including in unusual circumstances such as after missed appointment, loss of vaccination card and when the health workers failed to make home visits. Provider-client relationships are poor with mothers reporting fear of mistreatment and lack of cooperation from service providers. Contrary to what health workers and managers believe, mothers were knowledgeable about the benefits of vaccination. The high workload on mothers compounded by the lack of support from male partners was also found to contribute to the problem. Health system factors that contributed to the problem were poorly arranged and coordinated immunization services, vaccine and supplies stock outs, and lack of viable defaulter tracking systems in the health facilities.

**Conclusions:**

The main reasons for defaulting from the immunization program are poor counseling of mothers, unsupportive provider-client relationships, poor immunization service arrangements, and lack of systems for tracking defaulters. Efforts to reduce defaulter rates from the immunization program need to focus on improving counseling of mothers and strengthening the health systems, especially with regards to service arrangements and tracking of defaulters.

## Background

Reduction of mortality and morbidities from vaccine-preventable diseases in developing countries involves successfully implementing strategies that ensure high coverage and minimize drop-outs and missed opportunities [1]. Reports by the World Health Organization (WHO) and United Nations Children’s Fund (UNICEF) showed that an estimated two to three million deaths in all age groups are averted each year as a result of vaccinations against diphtheria, tetanus, pertussis, and measles [2]. In general, covering as many diseases as possible, ensuring vaccine potency and achieving high immunization coverage are essential requirements for childhood immunization to have the desired public health impact of decreasing mortality and morbidity, and possibly eliminating some of the vaccine-preventable diseases [1]. These conditions, however, are not usually met in many settings, especially resource-poor countries in sub-Saharan Africa, including Ethiopia [3–5]. As a result, a large number of children in these countries remain at risk for acquiring infectious diseases that can cause serious illness, disability or death [5].

Increasing immunization coverage involves two key elements: increasing access to immunization services and reducing dropout rates. While health systems in developing countries seem to improve access to immunization services steadily, the overall change in coverage remains suboptimal. In Ethiopia, for example, the administrative coverage of immunization showed significant improvement from as low as 42% in the 1990s to more than 88% in 2013, and the country has increased the number of diseases covered by the program from six in 1980 to ten in 2013 [Ethiopian national immunization coverage survey. 2012. Ethiopian Health and Nutrition Research Institute (EHNRI), Unpublished]. Ethiopia, however, falls short of achieving the target 95% of coverage for childhood immunization despite tremendous efforts by the government and its bilateral and multilateral partners. In analyzing the challenges to achieving high coverage in Ethiopia, it is evident that over the years, there have been virtually no achievements in reducing dropout rates. The 2011 Ethiopian Health and Demographic Survey (EDHS) report showed a first to the third dose dropout rate for Diphtheria – Pertussis - Tetanus (DPT) vaccine of 43% [3], indicating the lack of any improvements since 2005 when a similar survey found a DPT 3 dropout rate of 44% [4]. Comparison between the 2006 and 2012 national immunization coverage survey reports also shows the same trend of an increase in overall coverage over the six-year period with dropout rates remaining high [6]. Therefore, the persistently high dropout rate is certainly a major contributor to the suboptimal gains in immunization coverage.

Past studies found a variety of sociocultural and health system factors associated with incomplete immunization. These include caregiver literacy and employment [7–11], geographic location [7, 8, 12, 13], maternal utilization of other health services and place of childbirth [7, 14], safety concerns, and misconceptions about vaccines [15, 16]. Lack of awareness in the form of poor understanding of benefits, dosing and schedules [10, 12, 14, 17, 18], is also among the commonly reported reasons for defaulting from the program. A few studies also identified household income, child illness [19, 20], poor male partner involvement [15], and large family size [16] to be associated with defaulting. Incomplete immunization is also commonly linked to weaknesses in the health systems, such as the lack of vaccines during vaccination sessions [17] and poor geographic access and long distance from the sites [7, 8, 12, 15, 21], including in urban settings [13]. Poor service arrangements with long waiting time [13, 15], inconvenient schedules [16], unwelcoming health care environment [16, 19], and not holding sessions as planned [22] were also mentioned. Also, the lack of organized outreach services and functional defaulter tracing mechanisms have also been cited as contributing to incomplete immunization [22]. Provider related issues, such as poor counseling and relationship with health workers have also been described as reasons for incomplete immunization. Among the commonly reported gaps on counselling is not providing sufficient information on schedule, dosing and side effects [10, 17, 18].

It is, therefore, clear that a wide range of sociocultural, health systems and provider-related factors contribute to defaulting from immunization programs. It is also worth noting that high dropout rates continue to be among the biggest obstacles to achieving high coverage of vaccination in developing countries, hugely undermining gains obtained via improved access.

The aim of this study was to examine the reasons behind this persistent challenge. Although several studies presented here seem to have addressed the problem, nearly all are quantitative in nature with the issue of dropouts and reasons discussed as part of coverage surveys among children 12–23 months old. These have primarily generated a list of associated factors and reasons that did not seem to help health systems design effective strategies to address the problem. This study looked at the problem with a different approach with the aim to explore the issue in detail and help develop locally appropriate strategies to minimize defaulting from immunization programs. To this end, qualitative methods focused entirely on understanding why children default from immunization programs was conducted. Besides, the issue was explored among a younger age group (6–11 months) to help design early interventions and allow for a shorter recall time compared to 12–23 month-old children.

## Methods

### Study setting

The study was conducted between November 25, 2014 and April 24, 2015 in two districts of Hadiya zone in the Southern Nations Nationalities and Peoples’ Region of Ethiopia. The zone has a total projected population of 1.6 million distributed in 11 districts, which are further divided into 329 villages. Hadiya zone has one hospital, 61 health centers, and 305 health posts providing health services, including immunization. In line with the national strategies, the zone is providing Expanded Program on Immunization (EPI) services through static and outreach strategies. Static immunization services are provided at the health posts, health centers, and the hospital. There are outreach sites providing immunization services for selected communities living more than 5 km from the nearest health facility. The Zonal Health Department provides overall guidance on implementation of the EPI program, provides inputs to districts and conducts quarterly review meetings to assess the performance of districts. District Health Offices are directly involved in providing support to health facilities, including through supportive supervision and quarterly review meetings. According to the 2013/14 administrative report, EPI coverage of Hadiya zone is 98%. The coverage in the study districts, Hosanna town, and Anlemo district, is 49% and 98% respectively. Pentavalent 3 dropout rate for the zone during the same period was 8%. The rates for Hosanna town and Anlemo district were 12% and 10% respectively. It is clear from these figures that the reported administrative coverage has to be taken with caution as the figures from dropout rates and coverage of the services in the capital of the zone do not support the administrative coverage data.

### Study design

A Phenomenological study paradigm has been adopted to investigate reasons behind defaulting from immunization. A phenomenological study was suitable for this purpose as it allowed detailed investigation and understanding of the various issues contributing to defaulting from immunization services. Phenomenology describes the subjective reality of events as perceived by the study population and thus helps the researcher obtain the first person version of the story of interest [23].

### Study population

The study populations were mothers of children between 6 and 11 months that defaulted from vaccination, EPI focal persons of four health facilities selected from the two study districts, health extension workers from selected health posts in villages, immunization program managers at the zonal and district health offices and community leaders.

### Sample size

From the two study districts, a total of four health facilities were selected for the survey. Selection of the health institutions was made based on their location and dropout rates. To examine the issue in both urban and rural contexts, two of the facilities selected were urban while the remaining two were rural. After identification of all the urban and rural facilities in the two districts, selection of specific facilities was made based on their PENTAVALENT 3 defaulter rates in 2013/14. Two urban facilities and two rural facilities with the highest rates were included. Accordingly, from Anlemo district Bendelicho and Anagero health centers with PENTAVALENT 3 dropout rate of 11% and 12% respectively, were selected. From Hossana town, Lichamba Health Center, which had PENTAVALENT 3 dropout rate of 10%, and Bobicho Health Center with a rate of 18% were included. A total of 28 individuals were interviewed: fourteen mothers of defaulted children, two district health office EPI focal persons, one zonal EPI focal person, four health center EPI focal persons (one from each facility), four health extension workers (two urban health extension workers and two from rural villages in each district) and three community leaders from the area (one from urban and two from rural communities). While the number of mothers interviewed was decided based on data saturation, the rest was decided based on their role in providing as well as managing immunization service, and thus their understanding of the issues that may be contributing to defaulting from immunization programs. While the inclusion of the program managers, health workers, and community leaders was initially planned, the need to include health extension workers did arise at the time of data collection when initial findings indicated the need for obtaining their insight on the problem.

Except for mothers, all participants were selected purposively. In the case of the mothers, sampling was done using the following procedures. In the health centers, children aged 6–11 month that defaulted on pentavalent3 during the preceding one-year period were identified from the EPI registration book and all the available information on the address of the selected children was obtained from the registers. While it was possible to get basic address information for most of the children from the registers, additional information on the addresses of some children was obtained from client charts, health extension workers, and women’s health development army. A list of these children with the address information was produced, and data collection was started with the first child’s mother. None of the mothers invited for the interview declined to participate.

### Data collection procedure

The semi-structured interview guide was the instrument used for in-depth interviews with mothers and key-informants. After defining the research objectives and reviewing relevant literature, interview questions were developed in English, and later the final version was translated to local language (Amharic) for data collection. Data collection was conducted between December 28, 2014 and February, 28 2015. Before data collection, informed consent was obtained from each interviewee. Data collectors read a standard information sheet and consent form to each participant and all the selected individuals agreed verbally to participate in the study. Written consent was not sought as most participants were non-literate. All the information was tape-recorded and field notes were also taken for a backup as well as to capture issues that could not be achieved using tape-recording. Data collection was carried out by one of the investigators and a trained data collector who is familiar with qualitative data collection methods and EPI program. Open-ended questions were used in semi-structured conversational format and probing was done to explore the issues in-depth. Interviews were held at homes of mothers of defaulted children and workplaces of the key informants. Interviews lasted an average of 30 min. On the other hand, a pretest of the questionnaire was carried out in Shashago district health office and Shashogo health center, places that are far from the two study districts but with almost similar socio-demographic characteristics, EPI coverage, and PENTAVALENT 3 defaulter rates.

In addition to the interviews, the principal investigator conducted observations at the pediatrics outpatient departments of the four health centers. The observation was done for two consecutive mornings in each of the four health centers with the aim to assess issues related to immunization history taking, identification of missed opportunities and defaulters. No tools were developed for this purpose as the objective is limited to this one issue.

### Data management

Immediately after the interviews, handwritten field notes were expanded, labeled and were archived in chronological order. All recorded audiotapes of the interviews were transcribed in Amharic and were later translated to English. Expanded notes, transcripts, and archival information sheet and correspondence related to the data collection event were used for analysis.

### Data analysis procedure

Data analysis in this research was a continuous iterative process, which was conducted at all stages. Data were analyzed manually using thematic analysis [24]. After the expanded notes, audio transcripts, and other materials for analysis were produced, the principal investigator reviewed all the notes and transcripts and listened to the audios for familiarization with the data. The data were organized for subsequent detailed reading and analysis. All sensitive data were anonymized. Emerging themes were identified and provisional categories were developed with a thorough reading of the materials. Particular attention was given to exploring relationships between the different categories such as “knowledge” and “social factors.” The themes and categories were then refined and the final set used for presenting and discussing the findings. Excerpts from original transcripts, mainly quotes from key informant interviews, are included in the analysis. The study was approved by the Gondar University Institute of Public Health Ethics Committee.

## Results

### Participants

All the 14 mothers were asked open-ended questions regarding the exact reasons why their children defaulted from vaccination. The mean age of the mothers was 27 years, and all except one of the mothers had more than one child. The mean age of the infants was 8.3 months. All the children defaulted at DPT 3, missing the second and/or the third dose and are thus DPT 1–3 defaulters. Seven of the children were nine months or older, and thus, based on the country’s EPI schedule, have missed their measles vaccination as well.

Other key informants were also asked open-ended questions that are relevant to the work they do or their roles in the communities. A wide-range of topics on the broader issues that could affect access to and utilization of immunization services. Since none of the key informants had any objective and systematically collected information on the issue, they mainly provided perceived reasons for defaulting from immunization programs. Common themes that emerged from the discussions are presented below.

### Knowledge of benefits of immunization

Knowledge of mothers regarding the benefits of vaccination did not appear a significant determinant for defaulting from vaccination. All of the mothers reported knowing the benefits of vaccination, although none of them could state specific benefits or the diseases against which the vaccines protect their children. Nearly all the mothers expressed their belief that vaccines are good for the health and well-being of their children. Also, none of them stated they defaulted on the child’s vaccination because they did not think vaccines are beneficial, or that they have any alternatives to vaccination. On the contrary, the EPI focal persons from the health centers and health offices believed community members lacked in-depth understanding of benefits of vaccination, and that it is one of the reasons children default from the program. One of the stated “*the level of awareness in the community is low and mothers skipped immunization due to trivial issues such as minor side effects and being busy with other activities.*” The health extension workers also believe the level of understanding is low, and that they are not doing enough to address the problem: “*I don’t think people are aware of benefits of vaccination. Our community needs repeated education. However, I haven’t addressed the awareness creation as well as I would have liked because of community fatigue.*”

### Knowledge of vaccination schedules and service arrangements

A good understanding of schedules and service arrangements would be critical for several reasons. First, mothers would know exactly what to do when they missed appointments due to any challenges and would not think being unable to make it on the appointment date ends their hopes of having their children vaccinated. Second, mothers who received the services through one approach, for example, home visits, would know they could get the service through other means, such as from static services, when the health workers did not show up. A closer look at the responses of some of the mothers showed an apparent gap in this regard. Respondents missed appointments due to reasons such as traveling to other places on or around the appointment date for different reasons, including funerals and visiting relatives. Almost all of the mothers who gave these reasons believed it was not possible to obtain immunization services in other facilities nor they were aware of the fact that they could still get the services once they got back to their area. Some of the EPI focal persons also recognize travel as an important reason for defaulting, but could not explain the dynamics of how travel to other areas leads to defaulting. Similarly, mothers did not know what to do when health extension workers missed home visits for vaccination after giving earlier doses at home. One of the mothers who defaulted on her child’s vaccination stated she did so because “*the health extension worker failed to show up for a home visit after giving the earlier doses at home.*”

### Loss of vaccination card

Three of the mothers reported a loss of vaccination card as a reason for defaulting on their children’s immunization. The ways in which loss of vaccination card led to defaulting, however, was different for each one of the mothers. One of the mothers stated that the card served as a reminder to her and its loss led to her forgetting the date. She also felt to have the card was a requirement for getting the service: *“The vaccination card reminds me to vaccinate my child, and I thought it is also required for getting the service. So I thought if I go there without the card they will not provide the service to my child.”* The reason for the second mother was that she would not be sure of the appointment date without the card and also that she might not be treated well by health workers: *“I was afraid to go to the health center because I lost the vaccination card. I was not also sure about the appointment date. Besides, I was afraid the health workers could disappoint me.”* The third woman had talked to the health extension worker about the lost card but was told that he would not be provided the service without the card.

### Poor partner involvement and excessive workload on mothers

Some of the mothers interviewed mentioned being busy with household responsibilities to be the reason behind defaulting on child’s vaccination as a mother from Anagero village emphasized: *“the reason was that I was busy. In rural areas, most of the work burden is on mothers. I was too busy with housework like caring for children.”* The socio-cultural issues such as going to the market, attending social ceremonies, and being busy with housework influencing defaulting from vaccination was also a view shared by most of the EPI focal persons, both from health facilities and district offices, and the health extension workers.

Mothers who mentioned being busy as a reason were also asked if they had any support from other family members, including their partners on child health care related issues, including vaccination. They reiterated that caring for children, including the responsibility to take them to health facilities is entirely the women’s role in their culture. The fact that male partners are not involved in matters of child health was also a view shared by one of the health extension workers.

### Health systems and health care provider factors

A variety of health system and health care provider-related issues were cited by mothers and other key informants as reasons for defaulting from immunization services. The main ones are presented below.

#### Problems with vaccine supply and service arrangements

Mothers, health workers, and EPI focal persons raised interrupted vaccine supply as one of the reasons for defaulting from immunization program. One of the mothers stated that her child was not vaccinated despite taking him to the health facility on the appointment date, as there was no vaccine in the health center at the time: *“… on the third visit, I was told upon arrival that there was no vaccine in the health center. The health worker gave me a new appointment date, but I haven’t gone there until now.”* EPI focal persons at zone, districts and health facilities also recognize the fact that interruption in vaccine supply does occur due to various reasons, and that it could contribute to defaulting: *“There is a problem with vaccine supply in our health facilities. Interruptions do occur due to several reasons, including malfunctioning of refrigerators. This results in mothers not getting the service on the appointment dates and they may fail to come back for the service later.”*

Change of appointment dates due to vaccine stock outs is not the only problem with service provision. Two other problems with the services, long waiting times in the facilities and inflexible time arrangement for busy and working mothers were also cited by the participants. One of the mothers who defaulted on her child’s vaccination stated her frustration this way: *“I waited for five hours in the health center until the health workers came from the funeral but even after that nobody paid attention to my child’s vaccination and me. So I came back without getting the service.”* The lack of commitment by some health workers and the possible effect this could have on defaulting is also expressed by the zonal and one of the district-level EPI focal persons that stated some health workers canceled immunization sessions by themselves. One of the mothers also reported her child defaulted due to missed home visit by the health extension workers after previous doses were provided at home.

Other aspects of the immunization services cited mainly by EPI focal persons are the fact that static services provided at the facilities may be provided only on certain days of the week and that outreach services in the zone are weak. Interviews also revealed that one of the health centers provided immunization services only on Fridays, making it difficult for several mothers who could not take their children to health facilities on Friday. Our review of health institutions and zonal EPI reports also showed that outreach services, which are expected to be conducted in areas outside five kilometers radius from the nearest health facility, were not carried out for over a year. The EPI focal persons from the zone and districts also confirmed the apparent lack of outreach services in the area.

#### Poor counseling and client-provider relationships

Mothers who showed poor understanding of the program were asked about the advice they received from health workers regarding their children’s vaccination. Several of them stated the advice they received from health care providers was minimal and was mostly nothing more than the next appointment date. The lack of appropriate counseling and the associated frustration is evident in what one of the mothers stated: *“…. Other than the appointment date, they never told me anything about the type of vaccine, including for what reason they give the vaccines. They only tell us to have our children vaccinated. Sometimes they come to our home and provide vaccines to children without telling us what they gave to the children and why.”* One of the EPI focal persons also expressed concern regarding counseling by health workers stating, “*in some cases, health workers do not deliver all the necessary messages to mothers.”*

Regarding relationships, discussions with several mothers revealed that they were typically afraid of the health workers rather than consider them their supporters or care providers. Fear of mistreatment, especially when mothers failed to meet expectations such as keeping vaccination cards with them or attending sessions according to appointments, was frequently reported. *“I didn’t take my child to the health center because I thought they might not give her immunization because the appointment date has passed.”* Another mother who knew the schedule and considered taking her child to the health facility after missing appointment consciously avoided going to the HC because she “*feared mistreatment by health workers.*”

#### Poor referral linkages and tracing mechanisms

Observation conducted in the four health centers to see how the intra-facility and inter-facility referral systems work in terms of identifying missed opportunities and defaulted children showed that at pediatric outpatient departments, health workers did not always take full immunization and thus potentially failed to identify missed opportunities and defaulters. Regarding inter-facility referral, respondents were asked how the system works. Two of the EPI focal persons stated that the lack of clear mechanisms for intra-facility referrals for immunization services could have contributed to defaulting especially in urban areas. The reason they gave was that it is customary for mothers to go to their family to give birth and stay there for a couple of weeks. According to the participants, whether they initiate vaccination at the rural site or wait until they get back, referral issues can be problematic and could contribute to defaulting. Another scenario discussed about inter-facility referrals by zonal EPI focal person was the possibility of families moving to other areas. It is, however, worth mentioning that none of the mothers interviewed mentioned these issues as reasons for defaulting.

The WHO guidelines outline standard operating procedures and mechanisms for identifying defaulters from vaccination every month and tracing them so that they are brought back to the health facilities to complete their immunization series [25]. Review of defaulter tracing systems in the four health facilities revealed the lack of evidence that the activity was carried out at the time despite the health workers stating they traced defaulters. Besides, the activity was not documented. Interestingly, the health worker from one of the health centers opened up to discussions on tracing of defaulters and stated they did not usually conduct defaulter tracing “*except when there is “pressure from higher level*” due to the frequent rotation of staff at the unit.

## Discussion

This study explored the reasons for defaulting from immunization programs. The main reasons were: lack of in-depth understanding of vaccination schedule and confusion surrounding immunization service delivery, loss of vaccination card, and a variety of sociocultural and health systems and health care provider related issues.

Two sets of studies commonly addressed the problem of incomplete immunization. Most of the research in this area investigated the issue indirectly by identifying factors associated with low vaccination coverage or looking at predictors of complete immunizations [6, 8, 10, 12, 14]. These studies have looked at both non-immunization and incomplete immunization, usually by comparing those who received all the required vaccines with those who dropped out at some stage. Fewer studies examined incomplete vaccination separately [7, 15, 19, 20].

Lack of knowledge on the side of the mothers has been a commonly reported reason for incomplete immunization. The reported knowledge gaps include knowledge of benefits of vaccination [26], scheduling and number of sessions [12, 27–29] and age at which vaccination is started or completed [14, 30]. In some cases, knowledge is expressed in broad terms only, such as knowledge towards immunization [19, 31–33]. In this study, the mothers of defaulter children interviewed have knowledge about the benefit of vaccination. However, they lacked knowledge on the various operational aspects of the service, especially the schedules and options to obtain the services in case of missed appointment or lost vaccination card.

Interestingly, loss of vaccination card as a reason for incomplete immunization is not a frequently reported factor in the past. The dynamics of how it leads to dropouts is not also commonly discussed. The Ethiopian health system lacks clear guidelines on what to do when mothers show up for child vaccination without a card. Besides, whether vaccination cards are kept at home or in the health facilities also varied from place to place with only 29% of children 12–23 months of age found to have vaccination cards at home [3]. It is, however, clear from our findings that cards could be lost and, in combination with other factors, such as negative perceptions toward health workers and lack of knowledge on what to do under these circumstances, mothers failed to return to health facilities for child vaccination services or are actually denied the service when tried to seek help after a lost vaccination card.

Lack of empowerment of women, the excessive workload on mothers [16, 29], and poor paternal involvement [16] have previously been reported to be among the reasons for incomplete immunization among children. Two forms of maternal empowerment, education [8, 9, 12, 31, 34] and utilization of health services such as maternal tetanus vaccination [18, 28], having antenatal care follow up [14] and giving birth in a health facility [12, 14, 27] have been frequently reported as determinants or predictors of complete immunization. In this study, the high workload on mothers usually expressed as being busy was frequently mentioned and the lack of paternal involvement in matters of child health, including immunization were important contributors to defaulting from the program.

The World Health Organization identifies health system and health care provider related issues as the main contributors to incomplete immunization [35]. There are several areas in the system that could impact uptake of vaccination. Factors traditionally linked with the health system and are reported by some studies, such as distance [15] do not seem to be important in this study. However, the other commonly reported health system factors seem to contribute to defaulting from vaccination in this study hugely.

Due to inadequate counseling, mothers who took part in this study lacked clear and practical information on the scheduling, service arrangements, and options for vaccination in unusual situations such as when they lost vaccination card or missed appointment. Mothers also appeared to be confused about what to do when vaccination is initiated at home but health workers failed to visit for subsequent doses. Poor counseling, such as poor quality of information provided [27] and inadequate advice regarding the next appointment [36] have been found to contribute to the problem of incomplete vaccination in the past. Findings of this study are similar but provide more information about possible areas where the communication gaps occur.

In this study, mothers perceived health workers as unfriendly and not supportive. Thus in situations where they didn’t know what to do, such as when they lost vaccination cards or missed appointment, they anticipated to be mistreated by health workers, and typically avoided the health workers rather than consult them or seek support. Thus, mothers missed out on opportunities to get help and advice regarding how their children could get missed doses and even when they tried, they didn’t get a positive response from health workers. The relationship between perception of health professionals and the health institutions in general and completing immunization is previously reported from several developing countries, including Ethiopia [13, 15, 19, 26] and findings of this study also suggest mothers hold negative views about health workers and the treatment they get from them. These problems were also compounded by problems such as long waiting time and vaccines and supplies stock-outs, which were also mentioned previously [13, 15, 26].

This study found poor service arrangements as contributing factor to defaulting from immunization programs. Outreach services, home visits, and referral linkages are known to contribute to improvements in immunization coverage and minimizing defaulting and missed opportunities. This study found such activities were not regularly conducted and in fact, seem to have created confusion among mothers leading to defaulting of children from immunization.

The World Health Organization has provided guidance and tools for countries to have defaulter tracking mechanisms for immunization programs [25]. The defaulter tracking system works in such a way that children who defaulted are identified and recorded on a sheet and are traced and the outcomes of tracing are recorded. Ethiopia has adopted this system and the visited health centers also reporting conducting the listing and tracking of defaulters as in the WHO guidance. However, none of the health centers had any documented evidence of the activity, and there seems to be no structure at the health facility level to monitor this particular activity and ensure accountability. This lack of a viable defaulter tracking mechanisms in Ethiopia was also reported by a multi-country study conducted in 1999, and it seems that the surveyed health institutions were in the same situation. [22].

This study had several strengths, including examining the issue of defaulting at a younger age group (6–11 months) than was the case with many studies in the past. This approach helped to minimize recall bias and provided the chance for returning the defaulted children to the program. In addition, being a qualitative study, it was possible to examine the reasons behind defaulting in-depth, including the differences in what health workers and managers perceive and the actual reasons provided by the mothers. The failure to conduct a direct observation of the counseling provided to care givers at immunization units to substantiate the findings was the main limitation of the study.

## Conclusions

This study revealed some of the most important reasons why mothers default on their children’s vaccination and the gaps in the health system contributing to the problem. Contrary to what health workers and managers believed, mothers of defaulted children seem to be knowledgeable about the benefits of vaccination. However, their understanding of the schedule and the options for receiving vaccination services in unusual circumstances such as loss of vaccination card, missed appointment, and missed home visits by health workers was poor and seemed to be important reason children defaulted from the program. Mothers lacked information about schedule, sessions and service arrangements, and the non-supportive provider-client relationship made consultation of health workers and information seeking in these situations difficult. The most salient sociocultural issue contributing to defaulting from immunization programs is the lack of male partner involvement in child health matters, particularly vaccination. Poor counseling of mothers, unsupportive client-provider relationships, poorly arranged and coordinated immunization services, vaccines and supplies stock outs have also caused mothers to default on child vaccinations. The lack of functional immunization defaulter tracking mechanisms and poor intra-facility referral linkages were critical gaps in the health system.

Minimizing the number of defaulters from immunization programs requires the concerted efforts of all stakeholders. Counseling of mothers on vaccination should be standardized and strengthened and address key issues, including the schedules, service arrangements and commonly encountered circumstances that could prevent mothers from seeking the service. Mothers should be properly advised on the steps they need to take if they travelled to other locations, lost vaccination cards, children had side effects after immunization, or they missed appointment for any reason. Health workers need to be supportive of mothers. Awareness raising to empower women and increasing male involvement in child immunization are also important. Arrangements and coordination of immunization services need particular attention, especially in terms of coordinating static and outreach services to avoid possible confusion. Ensuring sustained supply of vaccines and instituting effective referral linkages and defaulter tracking mechanisms would also be vital. Defaulter tracing could be improved by leveraging the existing community structures, creating mechanisms for monitoring the activity, ensuring accountability, and learning from the rich experience of HIV care and treatment program.

## Abbreviations

DPT: Diphtheria – Pertussis - Tetanus
EDHS: Ethiopian demographic and health survey
EPI: Expanded programme on immunization
HepB: Hepatitis B
Hib: Haemophilus influenzae type B
UNICEF: United Nations Children’s Fund
WHO: World Health Organization.
